# Supplementary material for: Improving Newborn Resuscitation by Making Every Birth a Learning Event
Source: Children (Basel). 2021 Dec 16;8(12):1194. doi: 10.3390/children8121194 (PMC8700033; doi:10.3390/children8121194)
Supplement: Supplementary file 1 [file children-08-01194-s001.zip › children-1468442-supplementary.pdf]

Supplemental Table S1. Keywords used in searches for bedside learning strategies

**Bedside Learning Strategy**	**Keywords**
Visual display of resuscitation algorithm	Neonatal Resuscitation Program algorithm<br>Helping Babies Breathe algorithm<br>Helping Babies Breathe action plan<br>Newborn resuscitation cognitive aid
Peer-to-peer support	Peer support newborn resuscitation<br>Team training newborn resuscitation<br>Communication neonatal resuscitation program<br>Communication Helping Babies Breathe
Expert coaching	Coaching newborn resuscitation<br>Mentoring newborn resuscitation<br>Telemedicine newborn resuscitation
Automated guidance	Mobile health newborn resuscitation<br>Smartphone newborn resuscitation
Delivery room checklists	Newborn resuscitation delivery log<br>Helping Babies Breathe checklist<br>Helping Babies Breathe reflection<br>Newborn resuscitation reflection<br>Neonatal resuscitation self-reflection<br>Neonatal Resuscitation Program reflection<br>Newborn delivery log reflection<br>Quality improvement neonatal resuscitation LMIC<br>Quality improvement Helping Babies Breathe<br>Quality improvement Neonatal Resuscitation Program<br>Quality improvement self-reflection neonatal resuscitation
Audits	Helping Babies Breathe case reviews<br>Newborn resuscitation case reviews
Debriefing	Debriefing neonatal resuscitation<br>Debriefing newborn resuscitation<br>Debriefing LMIC<br>Helping Babies Breathe debriefing
